# The State of the Language: English Observed

**Published:** 1985-01

**Authors:** J. D. Davies

**Affiliations:** Department of Pathology University of Bristol


### Book Review

**THE STATE OF THE LANGUAGE: ENGLISH OBSERVED**
**P. Howard**
**Hamish Hamilton, London, 1984**
**pp. 180+xii, hardback. Price £8.95**

The author's informal portrait on the dust cover of this book is misleading. The women in our family think that he is a look-a-like 1930's comedian in braces. Appearances can be deceptive. Court comedian certainly. Pre-war no.

To most in medicine writing is a necessary evil. All of us have had to write school essays, entrance examinations, and set questions in order to pass undergraduate and later hurdles in professional life. On the way various papers, theses, and later, coerced contributions may too have taken their part. Day-to-day we write to our colleagues. Day-to-day we also have to read their letters. Very good for the lumber jacks and bad for trees but if my suspicions are correct, not so easy for us. There is however an ever sprouting forest of published guides to good behaviour in this field. Advice on avoidance of Civil Servicese,[1] good medical and scientific communication,[2] and for all I know, on how to write to your local medical journal, is on offer. That said, we act both as observers and participants in a changing world. In earlier times more leisure might (may?) have allowed more grammatical manuscript copy.

Changes certainly have come: first typewriters, manual and electric; thereafter photocopiers, word-processors and portable dictaphones. All this expensive new apparatus then got mixed up (sorry, I mean *interfaced*) with secretaries, hard and software, and the authors.

No wonder English is not as it used to be. Our own usage and acceptance is changing, . . . even if guiltily.

Take heart, the guilt and changes have not gone unnoticed in the wider world. Even only occasional readers of *The Times* will know of Philip Howard's contributions on the changing usage of words. He has formalised his personal observations in the form of a seemingly dictatorial series of chapter headings. In fact they are nothing of the kind. There is no Fowlerian prescription. Notes and sympathy for modern users of the dictaphone, telephone, handwritten foolscap and the word processor are there to help.

Everyone can feel easier. Past battles are described: how the apostrophe came to be; wobbles over *will* and *shall*; alternative spellings, and even an account of the origin of "as sick as a parrot". More and beyond.

No one, sender or recipient of letters, general reader or writer, could fail to be fascinated by this account of the current state of The English language. Fortunately most local booksellers stock this publication. Just walk in and ask.

J. D. Davies
Department of Pathology
University of Bristol

**REFERENCES**

1. FRASER, B. (1973) Gowers' The Complete Plain Words. London: HMSO. Revised edition.
2. THORNE, C. (1970) Better Medical Writing. London: Pitman. 2nd edition also available.

